# The invasive land flatworm *Arthurdendyus triangulatus* has repeated sequences in the mitogenome, extra-long cox2 gene and paralogous nuclear rRNA clusters

**DOI:** 10.1038/s41598-024-58600-y

**Published:** 2024-04-03

**Authors:** Romain Gastineau, Claude Lemieux, Monique Turmel, Christian Otis, Brian Boyle, Mathieu Coulis, Clément Gouraud, Brian Boag, Archie K. Murchie, Leigh Winsor, Jean-Lou Justine

**Affiliations:** 1 Institute of Marine and Environmental Sciences, University of Szczecin, Szczecin, Poland; 2 Département de Biochimie, de Microbiologie et de Bio-Informatique, Institut de Biologie Intégrative et des Systèmes, Université Laval, Quebec, QC Canada; 3 Plateforme d’Analyse Génomique, Institut de Biologie Intégrative et des Systèmes, Université Laval, Quebec, QC Canada; 4 CIRAD, UPR GECO, 97285 Le Lamentin, Martinique France; 5 GECO, CIRAD, University Montpellier, Montpellier, France; 6 UMR CNRS 6553 Ecobio, Université de Rennes, 263 Avenue du Gal Leclerc, CS 74205, CEDEX, 35042 Rennes, France; 7 The James Hutton Institute, Invergowrie, DD2 5DA Scotland; 8 Sustainable Agri-Food Sciences Division, Agri-Food and Biosciences Institute, Belfast, BT9 5PX Northern Ireland; 9 College of Science and Engineering, James Cook University of North Queensland, Townsville, QLD Australia; 10 ISYEB, Institut de Systématique, Évolution, Biodiversité (UMR7205 CNRS, EPHE, MNHN, UPMC, Université des Antilles), Muséum National d’Histoire Naturelle, CP 51, 55 Rue Buffon, 75231 Paris Cedex 05, France

**Keywords:** Arthurdendyus, Invasive flatworm, Mitogenome, Short- and long-reads sequencing, Tandem repeats, Paralogous rRNA, Mitochondrial genome, Biodiversity, Phylogenetics, Zoology

## Abstract

Using a combination of short- and long-reads sequencing, we were able to sequence the complete mitochondrial genome of the invasive ‘New Zealand flatworm’ *Arthurdendyus triangulatus* (Geoplanidae, Rhynchodeminae, Caenoplanini) and its two complete paralogous nuclear rRNA gene clusters. The mitogenome has a total length of 20,309 bp and contains repetitions that includes two types of tandem-repeats that could not be solved by short-reads sequencing. We also sequenced for the first time the mitogenomes of four species of *Caenoplana* (Caenoplanini). A maximum likelihood phylogeny associated *A. triangulatus* with the other Caenoplanini but *Parakontikia ventrolineata* and *Australopacifica atrata* were rejected from the Caenoplanini and associated instead with the Rhynchodemini, with *Platydemus manokwari*. It was found that the mitogenomes of all species of the subfamily Rhynchodeminae share several unusual structural features, including a very long *cox2* gene. This is the first time that the complete paralogous rRNA clusters, which differ in length, sequence and seemingly number of copies, were obtained for a Geoplanidae.

## Introduction

**Figure 1:**
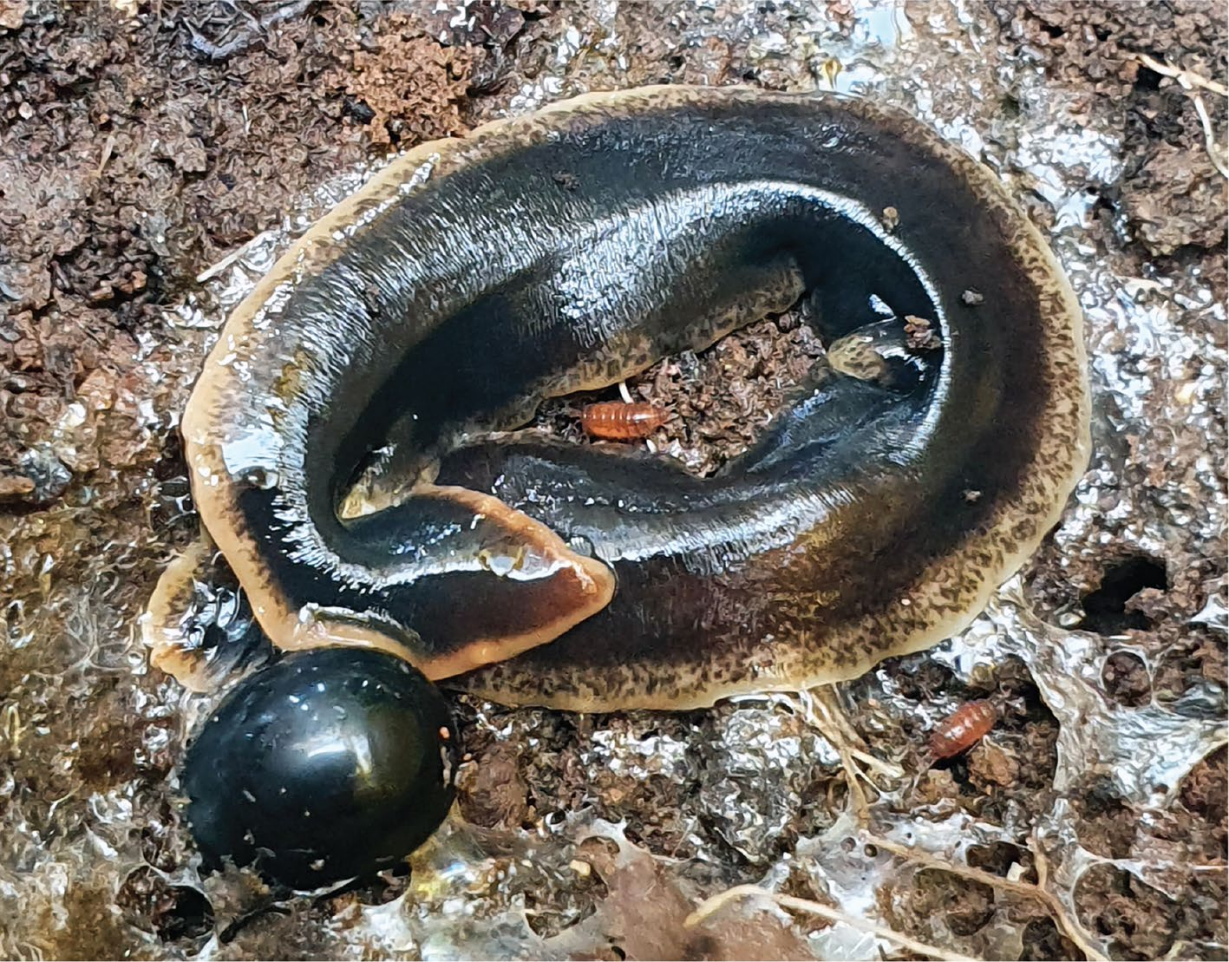
A live specimen of the invasive land flatworm *Arthurdendyus triangulatus* in a garden in Northern Ireland. Note the presence of a cocoon near the head. Unscaled—specimens typically measure 7–10 cm in length. Photograph by A. K. Murchie.

*Arthurdendyus triangulatus* (Dendy, 1894) is commonly referred to as the ‘New Zealand flatworm’, indicating its origin from the Southern Hemisphere (Fig. 1). This species of terrestrial flatworm (Geoplanidae) has earned a poor reputation as an invasive species and predator of native earthworms in north-western Europe^1^. Whether the dispersal of this species resulted from a single or several introductions is still debated^2,3^. Nevertheless, *A. triangulatus* is now well established in Great Britain and Ireland and has been recorded from as far as the remote Faroe Islands^4–9^. Because it develops well under temperate climates^10^, it has the potential to disperse among several other European countries^11,12^.

*Arthurdendyus triangulatus* is known for its predatory activity on lumbricid earthworms^9,13–16^. Given all the environmental consequences that this might have^17–20^, *A. triangulatus* has been included in the European list of Invasive Alien Species of Union concern. Transport or release of live specimens of *A. triangulatus* has thus been banned in the European Union to help prevent further dispersal.

*Arthurdendyus triangulatus* is not the only species of terrestrial flatworm that has become invasive in Europe and beyond^21–38^. Invading species of terrestrial flatworms are represented by several subfamilies of the Geoplanidae, among which some belong to the Rhynchodeminae, the subfamily that includes *A. triangulatus*^25,26,30,39^ within the tribe Caenoplanini. A similarly case concerns the genus *Caenoplana* Moseley, 1877 that now has species present in Europe, with probable unsuspected and underestimated biodiversity^21,40–44^. In Table 1, a summary of the currently accepted classification is provided.

**Table 1:** A simplified outline of the classification of the superfamily Geoplanoidea.

Superfamily GEOPLANOIDEA
Family DUGESIIDAE
Genus *Dugesia—*including* Dugesia constrictiva, D. japonica, D. ryukyuensis*
Genus *Girardia—*including *Girardia* sp*.*
Genus *Schmidtea—*including* Schmidtea mediterranea*
Family GEOPLANIDAE
Subfamily BIPALIINAE
Genus *Bipalium—*including* Bipalium kewense, B. vagum, B. adventitium, B. admarginatum*
Genus *Diversibipalium—*including* Diversibipalium multilineatum, D. mayottensis*
*Genus Vermiviatum—*including* Vermiviatum covidum*
Subfamily MICROPLANINAE
Genus *Microplana*
Subfamily RHYNCHODEMINAE
Tribe Rhynchodemini
Genus *Rhynchodemus*
Genus *Platydemus—*including* Platydemus manokwari*
Tribe Caenoplanini
Genus *Caenoplana—*including *Caenoplana variegata, C. coerulea, C. decolorata, Caenoplana* sp. “brown”
Genus *Arthurdendyus—*including* Arthurdendyus triangulatus*
Genus *Australopacifica—*including Australopacifica atrata*
Genus *Parakontikia—*including Parakontikia ventrolineata*
Tribe Anzoplanini
Genus *Anzoplana*
Tribe Eudoxiatopoplanini
Genus *Eudoxiatopoplana*
Tribe Pelmatoplanini
Genus *Pelmatoplana*
Subfamily GEOPLANINAE
Genus *Amaga—*including* Amaga expatria*
Genus *Obama—*including *Obama nungara*
Subfamily TIMYMINAE
Genus *Timyma*

Only some genera are listed, and only species with detailed data on the mitogenome are shown; however, all subfamilies and tribes are listed. Based on the phylogeny of Sluys et al.^45^ with addition of the newly described subfamily Timyminae^46^. The species formerly known as *Humbertium covidum* is mentioned here as *Vermiviatum covidum* following the recent reclassification of Bipaliinae (2023)^47^. All the species indicated are included in the multigene phylogeny presented below.

*Our results contradict the inclusion of *Australopacifica* and *Parakontikia* within the Caenoplanini; both are close to *Platydemus* (Rhynchodemini).

Since the pioneering work by Solà et al.^48^, the first to include the description of the complete mitogenome of a geoplanid, i.e., that of *Obama nungara* (Carbayo, Álvarez-Presas, Jones & Riutort, 2016), several other species have been similarly investigated phylogenomically^31,49–54^. Amongst these results, one peculiar feature that was noticed was the presence of an unusually long *cox2* gene among the three species of Rhynchodeminae studied^50–52^, namely *Platydemus manokwari* de Beauchamp, 1963, *Parakontikia ventrolineata* (Dendy, 1892) and *Australopacifica atrata* (Steel, 1897). To our knowledge, this feature has not been observed in other Metazoa. The extra-length in the *cox2* sequence has no known role, and beyond the three species already studied before this work, its distribution among Rhynchodeminae was unknown^50–52^.

Another peculiarity not restricted to Rhynchodeminae, but also observed in two families of the superfamily Geoplanoidea (Table 1), is the presence of two paralogous clusters encoding the nuclear rRNA genes^40,54–57^. Aside from representing a biological trait that deserves deeper studies, the existence of these divergent nuclear rRNA gene clusters may be problematic for molecular barcoding and phylogenetic analyses based on nuclear rRNA genes.

**Figure 2:**
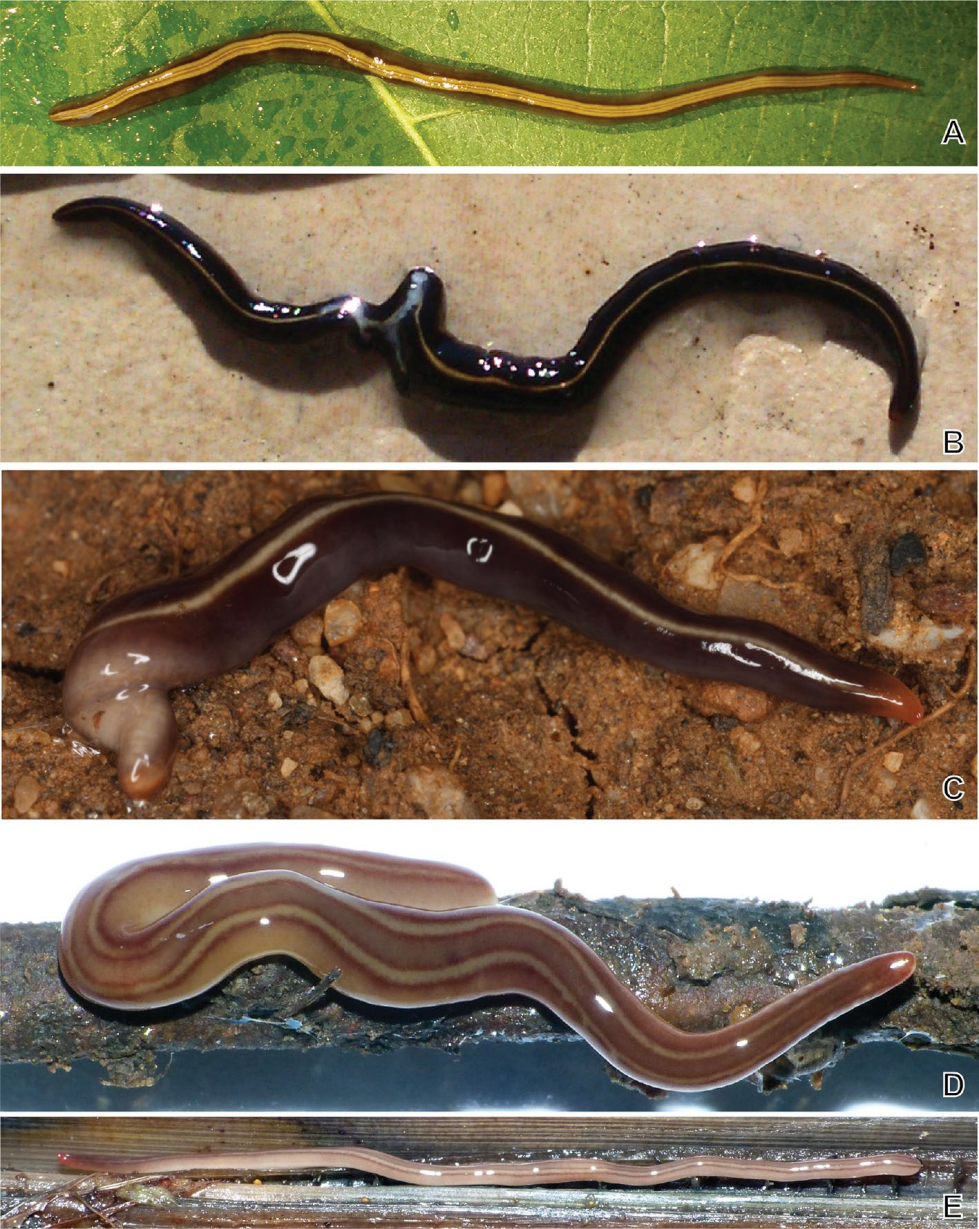
Live specimens of the four species of *Caenoplana* sequenced here. A, *Caenoplana variegata*, specimen MNHN JL144, hologenophore; photograph by Jean-Lou Justine. B, *Caenoplana coerulea*, specimen from the same population (same garden) as specimen MNHN JL194; photograph by Damien Michalski. C, *Caenoplana decolorata*, specimen PT426 illustrated in the original description of the species^42^; photograph by Eduardo Mateos. D, E, *Caenoplana* sp. “brown’, specimens from Martinique. D, specimen MNHN JL413, E, specimen MNHN JL399; photographs by Mathieu Coulis. All photographs, unscaled; live specimens measure 5–10 cm in length.

In the current study, we assembled the complete mitochondrial genome of *A. triangulatus* by using a combination of short- and long-reads sequencing technologies. Our data also enabled us to obtain for the first time the complete sequences of the two paralogous rRNA gene clusters for a geoplanid. The *A. triangulatus* mitochondrial genes were used to produce a molecular phylogeny that included four distinct species of *Caenoplana*, namely *Caenoplana variegata* (Fig. 2a), *Caenoplana coerulea* (Fig. 2b), *Caenoplana decolorata* (Fig. 2c) and *Caenoplana* sp. “brown’ (Fig. 2d), for which we also sequenced mitogenomes, although the completion of these genomes will be discussed later. In addition, the mitochondrial data were used for a broad comparison of the extra sequence present in the *cox2* gene.

## Results

### Assembling mitogenomes using short reads

For each of the five species examined, a large linear contig with all conserved mitochondrial genes was found following short-reads assemblies. In the case of *Caenoplana* spp., overlapping sequences were found at the ends of these contigs after their assembly or following their treatment with Consed, sometimes displaying polymorphisms or single nucleotide misalignments. Two interpretations are possible: (a) the mitogenomes of *Caenoplana* spp. are complete or (b) they might also contain several repeats at one or both of their ends, making their real sizes uncertain. In the case of *A. triangulatus*, the retrieved 15,716 bp contig after assembly showed no overlapping sequences at its ends; however, the use of the addSolexaReads.pl function of Consed in conjunction with data-mining of the contigs file led to the discovery of six small contigs that could be merged into a circular mitogenome of 18,059 bp. However, coverages of these contigs varied extensively, ranging from 66 to 282X. This suggested the presence of repetitions that cannot be resolved using short reads. As indicated in the Material and Methods, the 15,716 bp contig was later used as a database for filtering long reads.

### Processing the long-reads sequencing data

The basic statistics of the long-reads that were obtained before and after selection of the sequences specific to the mitogenome and nuclear rRNA gene clusters of *A. triangulatus* are indicated in Supplementary Table 1.

The assembly of the reads selected using the mitochondrial reference resulted in two contigs. The first one corresponded to the mitogenome; it was 20,281 bp long with a coverage of 40X and was detected by Flye as a sequence that can be circularised. The second contig was 752 bp long with a coverage of 10X and could not be identified. After the three iterations of Pilon and subsequent corrections, the final size of the mitogenome was 20,309 bp.

The assembly of the reads selected using the nuclear rRNA gene reference resulted in three contigs. The longest was 39,450 bp long with a coverage of 781X, followed by a 21,307 bp contig with a coverage of 141X. As reported below, our analysis indicated that they represent polymers of two different versions of the rDNA cluster. The last contig was 14,571 bp long with a coverage of 38X. Because Megablast queries showed that it belonged to an earthworm, probably from the genus *Eisenia* Malm, 1877, it was considered prey DNA.

### Characteristics of the *A. triangulatus* and Caenoplana spp. mitogenomes

**Figure 3:**
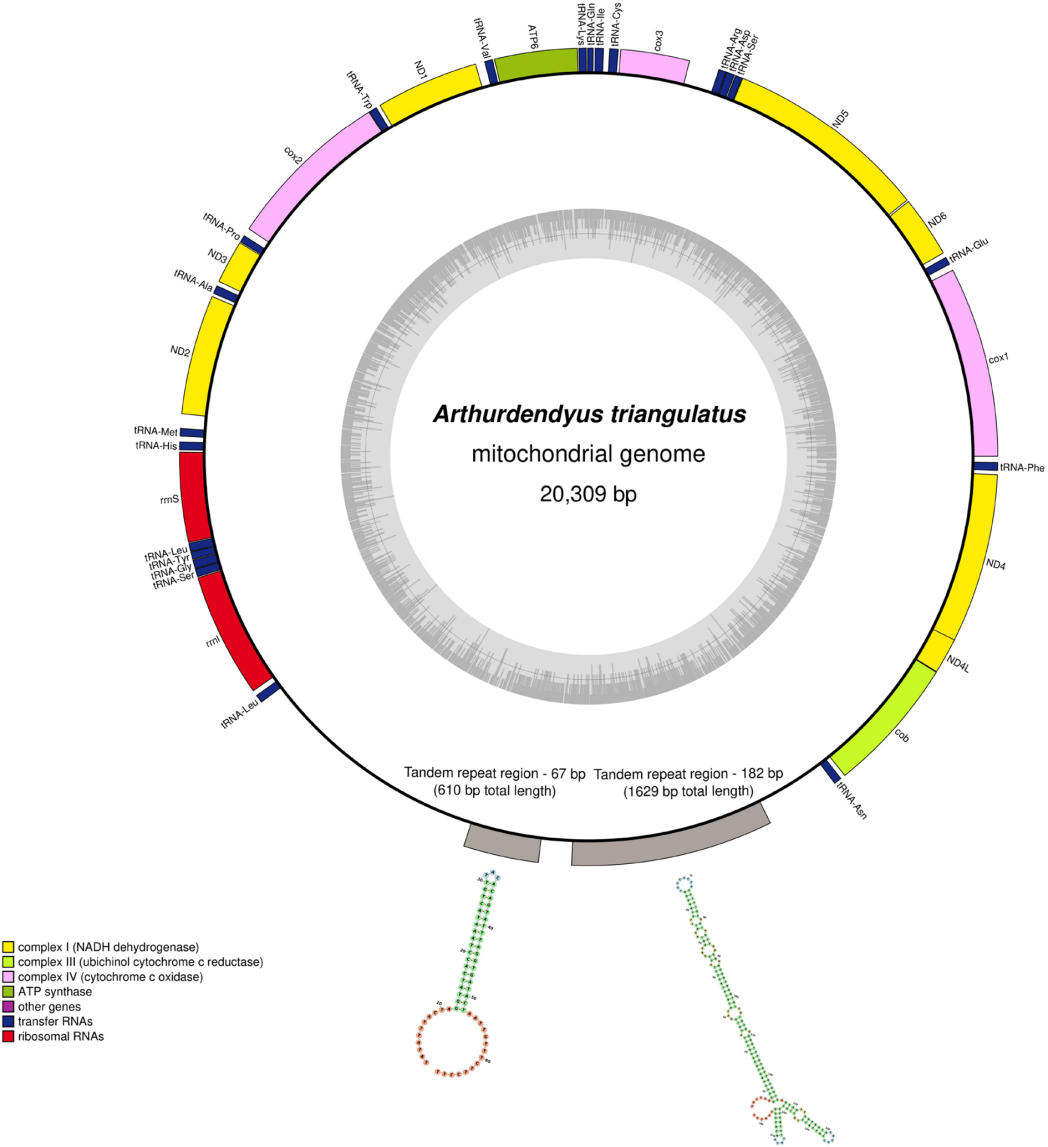
The mitogenome of *Arthurdendyus triangulatus* MNHN JL513. The colour boxes represent the different types of genes. The grey circle indicates the GC content. The grey boxes represent the extent of the two types of tandem repeats contained by the mitogenome. The position, length and secondary structure of the tandem repeats are indicated on the figure. The green colour of the dots indicates bases that could pair.

The five mitogenomes are all colinear with sizes ranging from 16,557 to 20,309 bp in size (Table 2), but as exemplified by our analyses of the *A. triangulatus* mitogenome, the genome sizes estimated for the *Caenoplana* spp. might have been underestimated. As suggested for *O. nungara*^48^ and illustrated here for *A. triangulatus*, geoplanids might display repetitions in their mitogenome that may not be resolved by short-reads sequencing. The cumulated length of all coding sequences in *A. triangulatus* is 14,336 bp, meaning that more than a quarter of the mitogenome is constituted by non-coding DNA. Using tandem repeat finder, we identified in the large non-coding part two conserved patterns with a noticeable number of repetitions. One has a consensus size of 67 bp with 98% match and was found 9 times. The second is longer and more conserved, being 182 bp long with 99% match and also present in 9 copies (Fig. 3).

**Table 2:** Accession number, size of the mitogenomes, sizes of the *cox2*-encoded protein and of their central elongation fragment for all species of Rhynchodeminae available.

Species	Accession number	Size of the mitogenome (bp)	Size of the *cox2* encoded protein	Size of the elongation fragment in the *cox2* encoded protein
*Arthurdendyus triangulatus*	OR835203	20,309	446 aa	146 aa
*Caenoplana variegata* JL144	OR835205	16,557	456 aa	148 aa
*Caenoplana decolorata* JL150	OR835204	17,722	458 aa	150 aa
*Caenoplana* sp. ‘brown’ JL410	OR835206	17,236	448 aa	147 aa
*Caenoplana coerulea* JL194	OR835207	18,621	505 aa	148 aa
*Platydemus manokwari*	MT081580	19,959	452 aa	147 aa
*Parakontikia ventrolineata*	MT081960	17,210	433 aa	142 aa
*Australopacifica atrata*	OM456243	16,513	434 aa	146 aa
*Bipalium kewense*	MK455837	15,666	225 aa	0 aa
*Obama nungara*	KP208777	14,909	259 aa	0 aa

The last two lines show *O. nungara* (Geoplaninae) and *B. kewense* (Bipaliinae) for comparison.

The specific features previously reported for the mitogenomes of Rhynchodeminae^52^ were all found among the mitogenomes of *A. triangulatus* and *Caenoplana* spp. They all display a 32-bp overlap between *ND4L* and *ND4*, the *ND5* gene is terminated by the presence of *tRNA-Ser,* and the *cox2* gene is of unusual length. As already observed for some other Geoplanidae^31,50–52^, no *tRNA-Thr* gene could be identified in the mitogenomes of the species studied here with the exception of *C. coerulea*. For this species, a D-Loop missing tRNA was found at a position congruent with other species in which this tRNA was found. As in Soo et al.^54^, it was possible to find the completely conserved **TGT** anticodon of a putative *tRNA-Thr* between the 16S rRNA gene and cytochrome b genes in the other species. However such tRNA would once again have a poorly conserved structure (no cloverleaf shape and missing D- and T-loops) and therefore was not annotated as such for any of these species. It is noteworthy that for *A. triangulatus*, it was possible to find two putative *tRNA-Phe* with a cloverleaf shape. One was found in a place congruent with all other geoplanids, which is between *ND4* and *cox1*. The second was found between *tRNA-Leu* and *tRNA-Asn*, which is where *tRNA-Thr* has been found among some other species. Pending further information, this second *tRNA-Phe* was not annotated.

### The extra sequence present in cox2

**Figure 4:**
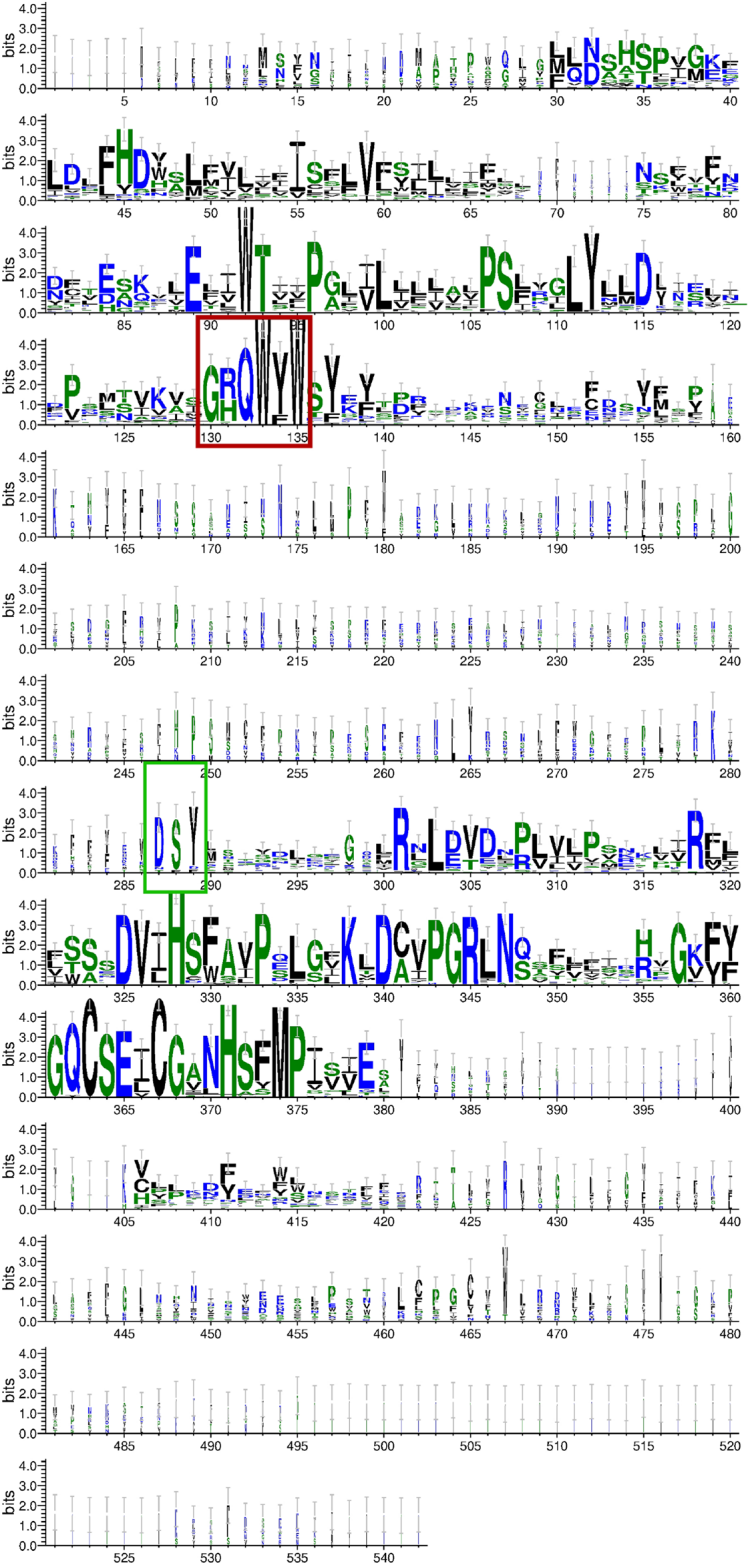
LOGO representation of the alignment of the Cox2 proteins obtained from several geoplanids and reference sequences downloaded from the Conserved Domains Database (all listed in Supplementary Table 2). The two coloured boxes indicate the conserved domains before (red box) and after (green box) the alignment breaks because of the extra-length displayed by rhynchodemins.

The amino acid alignment of the cytochrome c oxidase subunit II proteins is presented as LOGO (Fig. 4) The alignment stops and the expansion fragment starts after a conserved 6 amino acid pattern (surrounded by a red box in Fig. 4). The less-conserved region that follows, between positions 136 and 286, is due to substantial discrepancies in lengths and sequences in the Rhynchodeminae. The alignment resumes just before the C-terminal domain of the protein (highlighted by a green box on the figure), which contains, among others,the CuA binding site. It starts with a very conserved aspartate-serine dipeptide among all Geoplanidae, with a tyrosine residue mostly conserved thereafter.

**Figure 5:**
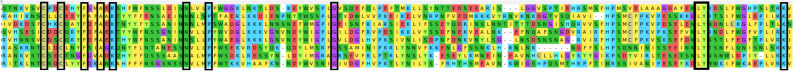
The extra-length in the Cox2 protein of rhynchodemins. The conserved amino-acid found among all taxa are indicated by rectangles. Screen capture of the alignment.

The alignment was trimmed to include only the amino-acid residues comprised between the hepta- and dipeptide described above (Fig. 5). The length of this region was calculated for each species plus *O. nungara* and *B. kewense* and the resulting values were found to be highly similar among the rhynchodemins, ranging from 142 to 150 amino acids (Table 2). Only 11 residues are conserved among all the eight species of Rhynchodeminae examined. From the N-terminal to C-terminal portion, they consist of two cysteine residues separated by two non-conserved amino-acids, a phenylalanine, an alanine, a lysine, an asparagine, a proline, a glycine, a leucine-tyrosine dipeptide and finally a lysine. It should be noted that the extra sequence in the middle region of *cox2* is not the only factor accounting for the greater length of the protein among rhynchodemins. The protein is also longer at the C-terminal part. Although this applies to all rhynchodemins, it is especially true for *C. coerulea*. There was no sign of a premature termination because of the presence of a tRNA, as opposed to the *ND5* gene for example. We are not ruling out a mistake of assembly that would have altered the canonical stop codon, but based on our software and sequencing data, we could not find evidence of this. Sequencing more specimens of *C. coerule*a should help to answer this question.

### The mitochondrial protein phylogeny

**Figure 6:**
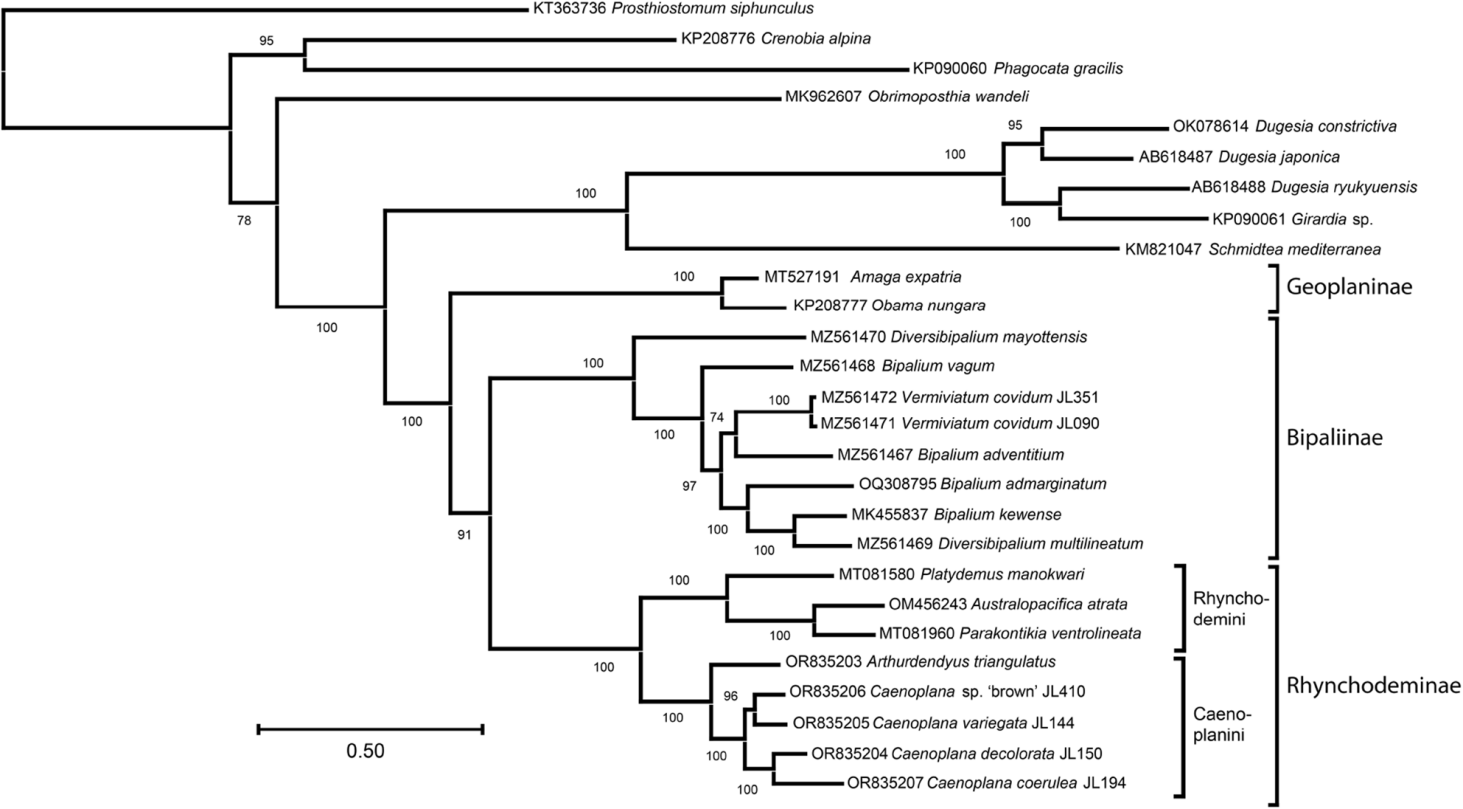
Maximum likelihood phylogenetic tree obtained from concatenated alignments of mitochondrial proteins using the MTZOA + I + G4 + F model of evolution and after 1,000 ultrafast bootstrap replicates. The names of subfamilies and tribes are indicated. *Arthurdendyus triangulatus* appears in a strongly supported clade with all species of *Caenoplana*. *Australopacifica atrata* and *Parakontikia ventrolineata* are rejected from this clade and instead associated with *Platydemus manokwari*.

The model of evolution returned by ModelTest-NG was the MTZOA + I + G4 + F for the llikelihood maximum (ML) phylogeny. The inferred phylogenetic tree revealed very high support at most of its nodes (Fig. 6). It unambiguously associates *A. triangulatus* with *Caenoplana* spp., but clearly distinguishes this clade from the other group of rhynchodemins represented by *Pl. manokwari*, *Pa. ventrolineata* and *Au. atrata*.

### Nuclear rRNA gene clusters

Using long-reads sequencing, it was possible to obtain the complete sequences of two paralogous nuclear rRNA gene clusters for *A. triangulatus*. Similarly to what was already suspected for *Bipalium admarginatum* de Beauchamp, 1933^54^, these clusters showed different coverages after assembly (Table 2), suggesting that their numbers of copies in the nuclear genome are noticeably different. When submitted to a Megablast analysis, the 18S rRNA gene version from the ‘high coverage’ cluster (HCC, OR797297) was found to correspond to type II (99.77% identity with AF033044), while the ‘low coverage’ cluster (LCC, OR797296) corresponded to type I (99.44% identity with AF033038). The sequence identity between the HCC and LCC versions of the 18S rRNA gene was 93.68%. For comparison, an alignment between the partial 18S genes of two different species of Geoplaninae, *Obama burmeisteri* (Schultze & Muller, 1857) Carbayo et al., 2013 (DQ666004) and *Obama anthropophila* Amaral, Leal-Zanchet & Carbayo, 2015 (KP962341)^58,59^ returned 96.13% identity, illustrating that the differences between two species might be lower than between the two clusters of the same species.

The two paralogous rRNA gene clusters of *A. triangulatus* also display distinct versions of the internal transcribed spacers 1 and 2 (ITS1 and ITS2), as well as distinct versions of the 5.8S rRNA gene. The sequence divergence between the ITS versions is substantial, with identities below 60%. It is also worth noting that the ITS1 size differs greatly between the two clusters (335 bp and 1207 bp for the LCC and HCC clusters, respectively). A difference in ITS length has also been observed for *Schmidtea mediterranea* Benazzi, Baguña, Ballester & del Papa, 1975^57^, the only species of the superfamily Geoplanoidea for which both versions of the ITS sequences were available prior to our study. All existing ITS references for *A. triangulatus*^2,3^ aligned with the HCC version of the ITS1-5.8S-ITS2 sequence. Note that the haplotypes detected in *A. triangulatus* by Roberts et al.^3^ should not be mistaken for HCC and LCC, as rather, they represent inter-individual variabilities.

In addition, there are two distinct versions of the 28S gene in *A. triangulatus* (Table 3). Their sequences differ at their 3’ ends. The two 28S gene sequences can align to a certain point, which corresponds to the 3’ end of the version found in the LCC cluster. Beyond this point, they diverge; however, when aligned against the reference sequence from *Mus musculus* (see below in Material & Methods), the 28S gene sequence present in the HCC cluster correctly aligns over a longer length (336 bp). To estimate the sequence identity between the two 28S genes, the gene sequence present in the HCC cluster was trimmed so that its 3’ end coincides with that found in the LCC cluster. The D2 variable region is especially poorly conserved, showing only 60.43% identity.

**Table 3:** Accession number and coverage obtained after assembly of the two paralogous clusters of nuclear rRNA genes in *A. triangulatus*.

	Accession	Coverage		18S	ITS1	5.8S	ITS2	28S
HCC	OR797297	781X	Size	1791 bp	335 bp	151 bp	426 bp	3476 bp
LCC	OR797296	141X		1791 bp	1207 bp	153 bp	510 bp	3151 bp
			Identity	93.68%	57.06%	94.70%	55.58%	89.29%

The size of its different components and the percentage of identity between paralogous clusters are also indicated.

## Discussion

With long-reads sequencing technologies becoming more widely available, it is expected that an increasing number of complex structures present in mitogenomes will be resolved, whether among vertebrates^60–62^ or invertebrates^63–69^.

In the current case, long-reads allowed us to resolve a nearly 5 kb long region between *rrnL* and *cob* containing two types of tandem repeats. Of particular interest for our study are the reports from Kinkar et al.^64–66^ and Oey et al.^67,68^. Using long-read sequencing methods, these authors found that Platyhelminthes such as the trematode *Schistosoma haematobium* (Bilharz, 1852), *Schistosoma bovis* Sonsino, 1876, *Clonorchis sinensis* (Cobbold, 1875) and *Paragonimus westermani* (Kerbert 1878) or the cestode *Echinococcus granulosus* Batsch, 1786 all display repeated regions in their mitogenome, with the most complex and longest repeated structure found in some strains of *S. haematobium.* In the latter, the length of the mitogenome ranges from 22.6 to 33.4 kb depending on the specimen^66^. There are, however, noticeable differences when compared with *A. triangulatus*. First, the position of the repeated region is different. It is located between *ND1* and *cox1* in *S. haematobium* and *S. bovis* and between *ND5* and *cox3* in *C. sinensis*. The number of tandem-repeat regions may also differ, as is exemplified by *E. granulosus*, in which two regions were found, one large between *ND5* and *cox3* as in *C. sinensis*, and one shorter between *ND5* and *ND6*. Secondly, the motifs of tandem-repeats might alternate as in *P. westermani* and *S. bovis*. Finally, in some cases, tRNA could be found between repeated motifs (e.g. *S. bovis*), which is not the case of *A. triangulatus.*

However, the above-mentioned organisms were not the first Platyhelminthes in which extra-long mitogenomes were investigated. Similar to our study, the work of Ross et al.^69^ on the 27,133 bp mitogenome of *S. mediterranea* uncovered the presence of a long non-coding region, but no mention of tandem-repeats was made in this publication. In this case also, the non-coding region was not resolved by long reads but rather by PCR amplification and Sanger sequencing. It is noteworthy that a 10-kb difference in the size of this non-coding region was observed between a sexual and an asexual specimen, the sexual specimen being the one displaying this extra length, which also contains a *tRNA-Ser* (missing from the asexual type). This order of magnitude compares with the observations done on the different specimen of *S. haematobium*. The non-coding region is located between *rrnS* and *ND2 in S. mediterranea*, which also differs from *A. triangulatus*.

Notably, the position of the *A. triangulatus* mitochondrial non-coding region that is associated with repeated structures, corresponds to a portion of the mitogenome of the geoplanid *Diversibipalium multilineatum* (Makino & Shirasawa, 1983) which we previously failed to circularise after assembly. It was indeed impossible to find overlapping sequences at its endings^31^. The presence of complex repeated structures in *D. multilineatum* could thus not be ruled out. Generally speaking, the presence, structure, and distribution of repeated sequences in mitogenomes among Geoplanidae is an open field for investigation. At present, it remains unknown as to which taxa contain mitochondrial repeats and whether these are conserved within a species or between closely related species. In the future, we may need to investigate some of these species again with long-reads technologies to verify our previous findings.

Thanks to the expanded alignment that we performed in the course of this study (Fig. 5), we could identify which conserved residues are present in the extension segment of the *cox2*-encoded protein of Rhynchodeminae. Introns in the mitogenomes of Metazoa are rather scarce, especially among bilaterians^70^, and we could not detect introns in the *cox2* gene. One of the explanations for the presence of this extra segment was that it could corresponds to an intein, i.e. a self-splicing element in the protein^71^. We therefore searched whether the conserved residues identified in the extra segment of Cox2 could represent conserved features of inteins as they are explicated in the InBase tool (https://inbase.ligsciss.com/iwai/InBase/tools.neb.com/inbase/index.html) based on the works of Perler^71,72^ and Pietrokovski^73^. The only conserved residues that could be found is a cysteine in what would be the predicted N-terminal splicing region and a serine in what would be the C-extein part. Nohistidine-asparagine or histidine-glutamine dipeptide was identified at the putative C-terminal splicing site. Inteins are rather scarce among Eukaryota and to our knowledge, none has been found in mitochondrial-encoded proteins^74^. In the absence of more convincing clues regarding the identity of the extra segment in the *cox2*-encoded protein of rhynchodemins, this segment should be considered an intrinsic component of the functional protein. Deeper investigations would require a proteomic approach to look for the presence of this extension in the mature protein, which is beyond the scope of the present work. Importantly, several reports indicate that there might be unusual initiation codons in the mitogenome of geoplanids, including rhynchodemins^48,53,75^. All these peculiarities advocate for more efforts in sequencing that should use long-reads sequencing as much as possible. It should also be noted that several tribes of Rhynchodeminae have not yet been sampled, namely Eudoxiatopoplanini Winsor, 2009, Anzoplanini Winsor, 2006 and Pelmatoplanini Ogren & Kawakatsu, 1991 (Table 1). An exploratory study on these organisms to look at the conservation of the *cox2* gene extra-length would thus be of interest.

Within the framework of this study, we obtained for the first time the two complete clusters of rRNA of a geoplanid. Several questions remain unanswered, and in some cases, routine protocols might be re-evaluated. The origin of these duplications remains unknown, and it is difficult to understand why both variants have been conserved. It is also unclear whether or not both clusters would be expressed and transcribed into functional rRNA. In their first publication on the topic, Carranza et al.^55^ had positive results only for the expression of the type I rRNA in *S. mediterranea*. However, in their second article on *Schmidtea polychroa* (Schmidt, 1861)^57^, they saw that both types might be expressed, although at very low levels for type II.

As noted above, the LCC rDNA cluster of *A. triangulatus* corresponds to type I while the HCC cluster corresponds to type II when comparing with results previously obtained on *S. polychroa* by Carranza et al*.*^56^. If the results of Carranza et al*.*^57^ on the expression of these two types were extrapolated to *A. triangulatus,* this would mean that the type associated with the highest coverage (thus, the highest number of copies) would be the least expressed, which is rather counter-intuitive. Technologies like RNAseq could be used to compare the coverage of both rDNA clusters in the genome with the coverages of the RNA they encode.

There are direct consequences of our new findings regarding the use of nuclear rRNA genes for barcoding and phylogenetic inference among Geoplanoidea. One can predict that the HCC/type II cluster has statistically more chance to be amplified and sequenced than the LCC/type I cluster. This would mean that the least expressed and possibly non-functional type would likely be the one amplified.

In case both variants were independently amplified on two specimens of the same species for which no reference is available, this would definitely be an issue in terms of molecular barcoding. This would be the case especially for the D2 region of the *28S* gene, for which there is substantial literature on a wide range of highly diverse Eukaryota^76–81^. Using this marker poses potential problems with the Geoplanidae as exemplified by the very low 60.43% identity between both variants in *A. triangulatus*. It could introduce a strong bias in any inferred phylogeny or lead to inaccurate taxonomic assessment when using molecular barcoding.

Another important issue raised by these paralogous clusters would be the use of the *18S* gene in the early detection of invasive flatworms in soil by the means of environmental DNA and metabarcoding. Such methods are often conducted on other Eukaryota by amplifying the V4 and V9 variable parts of the *18S* gene. With geoplanids, the protocol would preferably be adapted, or different barcodes (eg. the *cox1* gene) used.

The increasing availability of long-reads DNA sequencing technologies will make it possible to study paralogous rRNA gene clusters on more species of Geoplanidae and with larger sample sizes. With more complete sequences of the two types of nuclear rRNA gene clusters, their rate of evolution could be analysed. In addition, long-reads DNA sequencing of additional Geoplanidae would advance our knowledge about the distribution of repeats in the mitogenome. We hope to be able to go further in this direction in the near future.

The protocol used for phylogeny (concatenated amino-acid sequences) once again returned robust results. Based on the tree presented here, the inclusion of *Pa. ventrolineata* and *Au. atrata* in the Caenoplanini is not supported by this phylogenetic analysis, corroborating the results previously obtained by other teams^21^ from partial *cox1* and 28S genes. Instead, both species are associated with maximum support to the Rhynchodemini (*Pl. manokwari* ). As with previous phylogenies^31^, Geoplaninae and Bipaliinae appear as distinct, highly supported clusters. As already stated, many tribes of Rhynchodeminae remain to be sampled (Table 1), and in some cases (e.g. the genus *Anzoplana* Winsor, 2006), there is currently not a single sequence available in GenBank. How the results of such an upgraded phylogeny would articulate with the morphological classification is an exciting question we hope to address soon.

## Material and methods

### Biological material

The origins of the specimens used in the course of this study are reported below. All specimens were registered in the collections of the Muséum National d’Histoire Naturelle in Paris, France. All were killed by immersion in hot water or 95% ethanol.

*Arthurdendyus triangulatus*: five specimens collected on July 12, 2022, by Brian Boag; Birch Brae, Knapp, Inchture, Perthshire, PH14 9RN, Scotland; coordinates: N 56.47005205158123, W -3.1614500498816174. One specimen used for molecular analysis; four specimens deposited in MNHN under registration number MNHN JL513 (Fig. 1).

*Caenoplana variegata* (Fletcher & Hamilton, 1888): two specimens collected on May 6, 2014, by Dhyma Gomez; La-Plaine-Saint-Denis, Seine Saint Denis, Metropolitan France. Specimens kept in MNHN under registration number MNHN JL144, portion of body used for molecular analysis. The specimen in Fig. 2A is the hologenophore.

*Caenoplana coerulea* Moseley, 1877: one specimen collected on November 7, 2014, by Damien Michalski; Arles, Bouches-du-Rhône, Metropolitan France. Specimen deposited in MNHN under registration number MNHN JL194, portion of body used for molecular analysis. Note that Álvarez-Presas et al.^21^ have emphasized that *C. coerulea* is a species complex, based on their molecular work and information from one of us (LW). The *cox1* gene of our specimen is 100% identical to several sequences in GenBank which were attributed to *C. coerulea *sensu lato morphotype Ca1^21^. The specimen photographed in Fig. 2B is from the same population (same garden) as the hologenophore.

*Caenoplana decolorata* Mateos, Jones, Riutort, & Álvarez-Presas, 2020: one specimen collected on May 2, 2014, by Clément Gouraud; Nantes, Loire, Metropolitan France. Specimen deposited in MNHN under registration number MNHN JL150, portion of body used for molecular analysis. The species identification was confirmed on the basis of the *cox1* gene (GenBank: MW203125)^82^. Specimen not photographed. The specimen in Fig. 2C is specimen PT426 illustrated in the original description of the species^42^.

*Caenoplana* sp. “brown”: three specimens collected February 26, 2019, by Mathieu Coulis; Le Lamentin, Martinique, French West Indies; coordinates N 1420001, W-6012222. Specimens deposited in MNHN under registration number MNHN JL410, portion of body of one specimen used for molecular analysis. This species is currently unnamed but has been called “Brown-striped flatworm” in the 2020 Reference of Wildlife of tropical North Queensland; there are records of its presence in Martinique, Florida (USA), and Australia (Queensland); the species is believed to be a native of New Caledonia or New Guinea. The specimens illustrated in Fig. 2D,E are from the same locality and are deposited in the MNHN as MNHN JL399 and JL413, respectively.

### Short-reads sequencing and assembly

*Arthurdendyus triangulatus* was sequenced by the Genomic Analysis Platform (PAG) of the Institute of Integrative Biology and Systems at Laval University (Quebec, Canada) (https://www.ibis.ulaval.ca/en/services-2/genomic-analysis-platform/). In order to minimize contaminations from the digestive tract, tissues from longitudinal regions were separated with a scalpel. After having been frozen in liquid nitrogen, tissues were first shredded using a vibro-grinding device MM 400 (Retsch) and cells were then transferred in an Eppendorf tube containing 1.0 mL of lysis buffer prepared with 50 mM Tris–HCl pH 8.0, 200 mM NaCl, 20 mM EDTA, 2.0% SDS and 20 mg/mL proteinase K. The latter mixture was incubated at 65 °C for 30 min. An equal volume of CTAB buffer containing 50 mM Tris–HCl pH 8.0, 1.4 M NaCl, 20 mM EDTA, 2.0% CTAB, 1.0% PVP 40,000 was added to the lysate and incubation was pursued for an additional 30 min at 65 °C. The suspension was cooled down for a few minutes before 5 µL of RNase A (100 mg/mL) were added; it was incubated at room temperature for 20 min and then split in two tubes, the contents of which were extracted twice with an equal volume of chloroform: isoamyl alcohol (24:1). Finally, DNA was precipitated with two volumes of EtOH, dried and dissolved in 100 µL of TE buffer (10 mM Tris–HCl pH 8.0, 0.1 mM EDTA). A total amount of 20.4 µg DNA was recovered. The distribution of the size of fragments in the DNA preparation was determined using the Femto Pulse from Agilent (Santa Clara, CA, USA). The library was produced using 500 ng of DNA broken with a Covaris M220 (Covaris, Woburn MA, USA) and the NEBNext Ultra II DNA Library Prep Kit Illumina from New England Biolab (Ipswich, MA, USA). A total amount of ca. 40 million clean 150 bp paired-end reads was obtained from the NovaSeq 6000 platform of Génome Québec (https://www.genomequebec.com/).

The four specimens of *Caenoplana* spp. were sequenced at the Beijing Genomics Institute (BGI) (Shenzhen, China) on a DNBSEQ-G400 platform. Tissues were sent in 95% ethanol and DNA was extracted at the BGI facilities following an internal protocol. For *C. coerulea* and *C. variegata*, 60 million clean 100 bp paired-end reads were obtained per specimen. For *C. decolorata* and *Caenoplana* sp. ‘brown’, 40 million clean 150 bp paired-end reads were obtained per specimen.

For all five species, short reads were assembled using SPAdes 3.15.5^83^ and a k-mer of 85 for the 100 bp reads and a k-mer of 125 for the 150 bp reads. Consed^84^ was used to verify the terminal endings of the linear contigs corresponding to the mitogenome by using its ‘addSolexaReads.pl’ script.

### Long-reads sequencing and assembly

Long-reads sequencing of the *A. triangulatus* DNA preparation was performed at the PAG using the Oxford Nanopore Technology. First, 3 µg of genomic DNA were treated with the PacBio Short Read Eliminator (SRE_XS) Kit (Circulomics/PacBio, Menlo Park, CA, USA). A DNA library was then prepared using the SQK-LSK-109 Kit from Oxford Nanopore Technology (Oxford Nanopore, Littlemore, UK) and a fraction containing 700 ng of DNA was loaded onto a R9.4 MinION cell that had 1438 active pores. After 24 h of sequencing, the cell was washed with a nuclease solution, loaded with the remaining ca. 500 ng of DNA library, and sequencing was resumed for a total time of 72 h. Basic statistics of long reads were obtained from NanoStat^85^.

The reads presumably assigned to the mitogenome and to the nuclear rRNA gene clusters were selected using the mtblaster.py script (https://github.com/nidafra92/squirrel-project/blob/master/mtblaster.py). For the mitogenome, the reference sequence for this search was the contig containing all the conserved mitochondrial genes that was assembled from short reads. The Blast-based parameters were 90% identity and 1e^−150^ evalue, with maximum size of 35 kb. For the rRNA gene clusters, the reference consisted of the partial sequences of the 18S (AF033038) and 28S (DQ665953) rRNA genes of *A. triangulatus*, and the filtration parameters were also 90% identity and 1e^−150^ evalue, with a maximal size of 35 kb. The resulting sets of selected reads were assembled using Flye 2.9.1^86^ with the—meta option and overlap parameter of 3000 for the mitogenome and 10,000 for the rRNA gene clusters. In the case of the mitogenome, the assembly was submitted to three iterations of Pilon 1.24^87^ using the pool of short reads previously obtained.

### Annotation of mitogenomes

All mitochondrial genes were annotated with the help of MITOS^88^ followed by manual curation, using the genetic code 9, except for the rRNA genes whose termini were mapped using alignments against published homologs. Positions of the tRNA genes were verified with Arwen v.1.2^89^. Repeats in the *A. triangulatus* mitogenome were analysed using Tandem repeats finder^90^ and Microsatellite repeats finder (http://insilico.ehu.es/mini_tools/microsatellites/?info). Tandem repeats were drawn as explained in Kinkar et al.^65^. Mitogenome maps were drawn with OGDRAW^91^.

### Annotation of nuclear rRNA gene clusters

Boundaries of the two rRNA gene clusters of *A. triangulatus* were determined using Rfam^92^. In the case of the 28S rRNA gene, alignments with the reference sequence of *Mus musculus* Linnaeus, 1758 (NR_003279)^93^ were required.

### Alignment of Cox2 proteins

The amino-acid sequences of the predicted proteins encoded by the *cox2* genes of *A. triangulatus* and *Caenoplana* spp. were aligned with the corresponding sequences of other species of Geoplanidae, Platyhelminthes, and reference sequences from the Conserved Domains Database (https://www.ncbi.nlm.nih.gov/cdd). All accession numbers are listed in Supplementary Table 2. The alignment was performed with MEGA11^94^. LOGO alignment was done on the online LOGO website (https://weblogo.threeplusone.com/).

### Phylogenetic analysis

The dataset previously used to infer a phylogeny of the Geoplanidae^31,54^ based on 21 mitogenome-encoded proteins was appended with the five new species examined here plus the recently described *Dugesia constrictiva* Chen & Dong, sp. nov.^95^. The amino-acid sequences of the individual proteins were first aligned using MAFFT 7^96^ and trimmed with the -automated1 option of trimAl^97^; then, the different protein alignments were concatenated using Phyutility 2.7.1^98^. ModelTest-NG v0.1.7^99^ was used to select the best model of evolution, with default option for maximum likelihood inference (ML). The ML phylogenetic analysis was performed using IQ-TREE 2.2.0^100^ and 1000 ultrafast bootstrap replicates.

### Supplementary Information

Supplementary Table 1.

Supplementary Table 2.

## Data Availability

The mitochondrial genomes are available on Zenodo as fasta and tbl files following this link: https://doi.org/10.5281/zenodo.10256232. All sequences have been deposited on GenBank with the accession numbers indicated in the text.
