# Supplementary material for: The invasive land flatworm Arthurdendyus triangulatus has repeated sequences in the mitogenome, extra-long cox2 gene and paralogous nuclear rRNA clusters
Source: Sci Rep. 2024 Apr 3;14:7840. doi: 10.1038/s41598-024-58600-y (PMC10991399; doi:10.1038/s41598-024-58600-y)
Supplement: Supplementary file 1 — Supplementary Table 1. [file 41598_2024_58600_MOESM1_ESM.docx]

|  | Number of reads | Number of bases | Mean read length | N50 | Mean quality |
| --- | --- | --- | --- | --- | --- |
| Pool before filtration | 1479158 | 2955700068.0 | 1998.2 | 3569.0 | 14.9 |
| Filtration on the mitochondrial reference | 6422 | 21380126.0 | 3329.2 | 5457.0 | 15.5 |
| Filtration on the rRNA reference | 15809 | 58245417.0 | 3684.3 | 5346.0 | 14.6 |

Supplementary Table 1. Statistics of the ONT reads obtained on Arthurdendyus triangulatus before and after filtration on references.
