# Supplementary material for: The invasive land flatworm Arthurdendyus triangulatus has repeated sequences in the mitogenome, extra-long cox2 gene and paralogous nuclear rRNA clusters
Source: Sci Rep. 2024 Apr 3;14:7840. doi: 10.1038/s41598-024-58600-y (PMC10991399; doi:10.1038/s41598-024-58600-y)
Supplement: Supplementary file 2 — Supplementary Table 2. [file 41598_2024_58600_MOESM2_ESM.docx]

| **Name** | **Accession number** |
| --- | --- |
| *Obama* sp. | AKC99403 |
| *Bipalium kewense* | QGI24380 |
| *Bipalium adventitium* | UHA56270 |
| *Diversibipalium mayottensis* | UHA56306 |
| *Bipalium vagum* | UHA56282 |
| *Diversibipalium multilineatum* | UHA56297 |
| *Humbertium covidum* | UHA56318 and UHA56330 |
| *Biplaium admarginatum* | OQ308795 |
| *Australopacifica atrata* | UZA66415 |
| *Dugesia ryukyuensis* | BAL41009 |
| *Dugesia japonica* | BAL40998 |
| *Dugesia constrictiva* | OK078614 |
| *Crenobia alpina* | AJO61386 |
| *Platydemus manokwari* | UKA77688 |
| *Parakontikia ventrolineata* | UKA77700 |
| *Amaga expatria* | QSM34663 |
| *Schmidtea mediterranea* | AFQ93455 |
| *Schistosoma mansoni* | AAG13158 |
| *Bos taurus* | gi\|40889824 |
| *Gallus gallus* | gi\|117013 |
| *Oryzias latipes* | gi\|82243222 |
| *Sus scrofa* | gi\|1706060 |
| *Macaca mulatta* | gi\|1706051 |
| *Paracoccus denitrificans PD1222* | gi\|119377621 |
| *Candida glabrata CBS138* | gi\|54040793 |
| *Pneumocystis murina* | gi\|452849342 |
| *Neurospora crassa OR74A* | gi\|363548471 |
| *Aspergillus nidulans FGSC A4* | gi\|378927074 |
| *Strongylocentrotus purpuratus* | gi\|117046 |
| *Schizosaccharomyces pombe* 972h- | gi\|19857059 |
| *Paracoccus denitrificans* | gi\|6730477 |
| *Homo sapiens* | YP_003024029.1 |

Supplementary Table 2. GenBank accession number of the reference Cox2 amino-acid sequences used for alignment.
